# Characteristics of fall recurrence among persistent, quasi-persistent, and transient fallers: a 3-year analysis from the SCOPE study

**DOI:** 10.1007/s41999-026-01555-2

**Published:** 2026-07-07

**Authors:** Itshak Melzer, Ellen Freiberger, Sabine Britting, Fabrizia Lattanzio, Andrea Corsonello, Yehudit Melzer, Rada Artzi-Medvedik, Regina Roller-Wirnsberger, Gerhard Wirnsberger, Francesco Mattace-Raso, Lisanne Tap, Pedro Gil, Francesc Formiga, Rafael Moreno-González, Tomasz Kostka, Agnieszka Guligowska, Johan Arnlov, Axel C. Carlsson, Paolo Fabbietti, Robert Kob

**Affiliations:** 1 Department of Physical Therapy, Recanati School for Community Health Professions at the faculty of Health Sciences, Ben-Gurion University of the Negev, Beer-sheva, Israel; 2 Department of Internal Medicine-Geriatrics, Institute for Biomedicine of Aging (IBA), Friedrich-Alexander-Universität Erlangen-Nürnberg, Kobergerstr. 60, 90408 Nürnberg, Germany; 3 Italian National Research Center on Aging (IRCCS INRCA), via S. Margherita 5, 60121 Ancona, Fermo and Cosenza, Italy; 4 Maccabi Health Organization, Negev, Israel; 5 Department of Internal Medicine, Medical University of Graz, Graz, Austria; 6 Department of Internal Medicine, Division of Nephrology, Medical University of Graz, Graz, Austria; 7 Department of Internal Medicine, Section of Geriatric Medicine, Erasmus MC, University Medical Center Rotterdam, Rotterdam, The Netherlands; 8 Faculty of Medicine, Département of Medicine Pza. Ramón y Cajal, s/n, Ciudad Universitaria, 28040 Madrid, Spain; 9 Geriatric Unit, Internal Medicine Department, Bellvitge University Hospital – IDIBELL – L’Hospitalet de Llobregat, Barcelona, Spain; 10 Department of Geriatrics, Healthy Ageing Research Centre, Medical University of Lodz, Lodz, Poland; 11 Department of Medical Sciences, Uppsala University, Uppsala, Sweden; 12 School of Health and Social Studies, Dalarna University, Falun, Sweden; 13 Department of Neurobiology, Care Sciences and Society, Division of Family Medicine and Primary Care, Karolinska Institute, Huddinge, Sweden; 14 Academic Primary Health Care Centre, Region Stockholm, Stockholm, Sweden; 15 Department of Nursing Sciences, Recanati School for Community Health Professions, Faculty of Health Sciences, Ben-Gurion University of the Negev, Beer-Sheva, Israel; 16 Present Address: Department of Nursing Sciences, Tel Aviv University, Tel Aviv, Israel

**Keywords:** Falls, Fall circumstances, Community-dwelling old adults, Longitudinal study

## Abstract

**Methods:**

To address the substantial disability and healthcare burden associated with falls in older adults, the aim of this study is to use a new 3-year operationalization of fall occurrence profiles to identify factors linked with persistent falling. Data were obtained from the multicenter SCOPE study, which followed 2461 community-dwelling adults (median age 80.0 and IQR 6.0; 56.1% women) across seven European countries, with assessments at baseline and after 1 and 2 years. Variables included demographics, comorbidities, medication use, functional status, mood, quality of life, and kidney function. Fall-related data included number, location, cause, and consequences. Participants were classified as Persistent-Fallers (falls at all three timepoints), Quasi Persistent-Fallers (falls in two), and Transient-Fallers (one fall episode). Logistic regression assessed predictors of being Persistent-Fallers vs. Transient-Fallers, and vs. Quasi Persistent-Fallers.

**Results:**

Across the study, 894 falls occurred in Persistent-Fallers (*n* = 137), 919 in Quasi-Persistent Fallers (*n* = 299), and 695 in Transient-Fallers (*n* = 524). Most falls occur outdoors and during ambulation. Persistent-Fallers showed poorer physical performance, worse health, and higher proportion of indoor falls. Transient-Fallers were more likely to fall outdoors but sustain a higher risk of fractures per fall. Quasi-Persistent Fallers displayed intermediate characteristics. In fully adjusted models, higher comorbidity burden, presence of lower urinary tract symptoms and poorer self-rated health were significantly associated with persistent falling.

**Conclusions:**

This study reveals distinct faller profiles. Persistent-Fallers reflect frailty and linked to overall medical burden and functional decline; Transient-Fallers are generally healthier, fall outdoors more often, and their falls more often lead to fractures, possibly due to lower risk awareness falls; Quasi-Persistent Fallers exhibit characteristics that fall between those of the other two groups.

## Key Summary Points

**Aim:**

To classify older adults into three fall profile groups over 3 years and identify factors linked with persistent falling.

**Findings:**

Persistent-Fallers were frailer, had poorer health, and more indoor falls. Transient-Fallers were healthier, fell mostly outdoors, and had a higher probability that a fall leads to a fractures. Quasi-Persistent Fallers showed intermediate features. Higher comorbidity, lower urinary tract symptoms, and poor self-rated health predicted persistent falling.

**Message:**

Older adults show distinct fall patterns. Recognizing these profiles can guide more targeted and effective fall prevention strategies.

## Introduction

Falls are a serious and costly health threat affecting older adults, with consequences ranging from minor injuries to long-term disability and death [1–3]. Falls are one of the leading causes of injury-related hospitalizations among people aged 65 and older, often resulting in fractures, and a steep decline in functional independence [4, 5]. In addition, many older adults who fall experience subsequent concerns about falling (CaF) that can lead to activity restriction, loss of autonomy, and social withdrawal [6]. For many years, this psychological condition was named fear of falling. However, the World Falls Guidelines (WFG) recommend using CaF instead, since this term avoids emotional connotations and misunderstandings with anxiety disorders [7]. These psychosocial consequences often set off a negative spiral, further increasing fall risk [8]. Falls among older adults pose a significant financial burden, for example, costing nearly USD 50 billion annually in the U.S. [5].

Despite extensive literature aiming to identify risk factors, such as balance impairment, polypharmacy, and environmental hazards [2, 9–11], there is a limited understanding of the longitudinal occurrence of falling in the community-dwelling older population. Most studies categorize fallers based on short-term or retrospective reports, often using binary definitions that do not capture the variability in fall occurrence over time [12–21]. Furthermore, while much of the above research has examined the location and acute causes of falls [12–18], less attention has been given to how fall occurrences evolve over multiple years, and what this might indicate about underlying risk profiles.

To address this gap, a recent study [22] examined characteristics of fall events, locations, activities, and consequences among older adults living independently in the community. While informative, this prior work focused on individual fall events rather than long-term fall profiles. Instead, we propose a new operationalization to distinguish between three subgroups: (1) Persistent Fallers (*P-Fallers*), who reported at least one fall during each of the three yearly data collection points (T0, T1, and T2); (2) Quasi-Persistent Fallers (*Q-Fallers*), who reported falls in two out of the three timepoints; and (3) Transient Fallers (*T-Fallers*), who experienced a fall in only one of the three timepoints (T0, T1, and T2). To our knowledge, no previous study has systematically compared these temporal fall occurrences. This nuanced typology may provide a more dynamic understanding of fall risk than conventional single-point-in-time approaches. Accordingly, we aimed to investigate the medical, functional, and fall circumstances and characteristics associated with each group of Persistent, Quasi-Persistent, and Transient Fallers, with the goal of contributing a novel perspective on fall risk heterogeneity among community-dwelling older adults. Our approach is also related to the Weighted Fall Probability (WFP) algorithm [7], which estimates an individual’s likelihood of future falls by integrating multiple risk factors into a composite probability score.

## Methods

### Study population

The Screening for Chronic Kidney Disease among Older People across Europe (SCOPE) study (European grant agreement No. 436849) is a multicenter, prospective cohort study that followed 2461 community-dwelling older adults aged 75 years and above at baseline (T0), after a year (T1) and after 2 years (T2). Recruitment took place across several institutions in Austria, Germany, Israel, Italy, the Netherlands, Poland, and Spain. A detailed description of the SCOPE study’s methodology has been published previously [23]. The study received ethical approval from all participating institutions and adhered to the principles outlined in the Declaration of Helsinki and Good Clinical Practice guidelines. All participants provided written informed consent before enrollment.

### Study protocol

Participants were invited to an assessment visit at the study centers over 3 years at baseline (T0), 1-year follow-up (T1), and 2-year follow-up (T2). Information on fall events in the past year and their circumstances was collected. We used the opportunistic case finding question also recommended by the WFG with the additional request about the number of falls: “How many times have you fallen in the past 12 months?”. Falls were defined as "the consequence of movement unintentionally and unexpectedly to the ground from a higher position" [22, 23]. The definition used in the SCOPE project differ slightly from the definition presented by the former PROFANE group missing the aspect of a “lower level” [24]. During structured interviews, participants provided the number of falls experienced in the previous year and details for each fall based on predefined response options. Herby, participants described the activity they were engaged in at the time of the fall, such as ambulation (walking, turning, standing), transferring (e.g., getting in or out of a chair or using a toilet), running, sports activities, or stair and curb navigation. For each fall, they were asked about the location (i.e., inside the home, outside the home but inside a building, or outdoors) and the perceived cause (i.e. trips, slips, or collisions; collapse episodes; dizziness or vertigo; or balance and gait impairments). Fall-related injuries were obtained from medical records and categorized as bone fractures, treated injuries, untreated injuries, or no injuries. In addition, concerns about falling were assessed using a single-item question, with responses ranging from "not at all concerned", “a bit concerned”, “somewhat concerned” to "very concerned. In this study, we analyzed the proportion of participants who reported not to be concerned to fall at all.

In addition, the assessments included demographic information, physical and clinical evaluations, number of comorbid conditions, medication use, living arrangements, and other relevant factors (see details in reference [23]). Functional status was measured using the Basic and Instrumental Activities of Daily Living scales (bADL and iADL), the Short Physical Performance Battery (SPPB) and Handgrip strength. Handgrip strength was measured, using a hydraulic grip strength dynamometer (Model J00105 JAMAR Hydraulic Hand, Lafayette Instrument Company, USA). Participants were encouraged to squeeze as hard as they could, 3 attempts were conducted for each hand alternating sides. The maximum strength of the dominant hand is reported in this manuscript. Additional evaluations included the 15-item Geriatric Depression Scale (GDS), the Cumulative Illness Rating Scale (CIRS) and lower urinary tract symptoms (LUTS). Quality of life was measured using the EuroQol 5D index scores. Blood and urine samples were also obtained. Estimated glomerular filtration rate (eGFR) was calculated using the Berlin Initiative Study (BIS) equation.

**Fig. 1:**
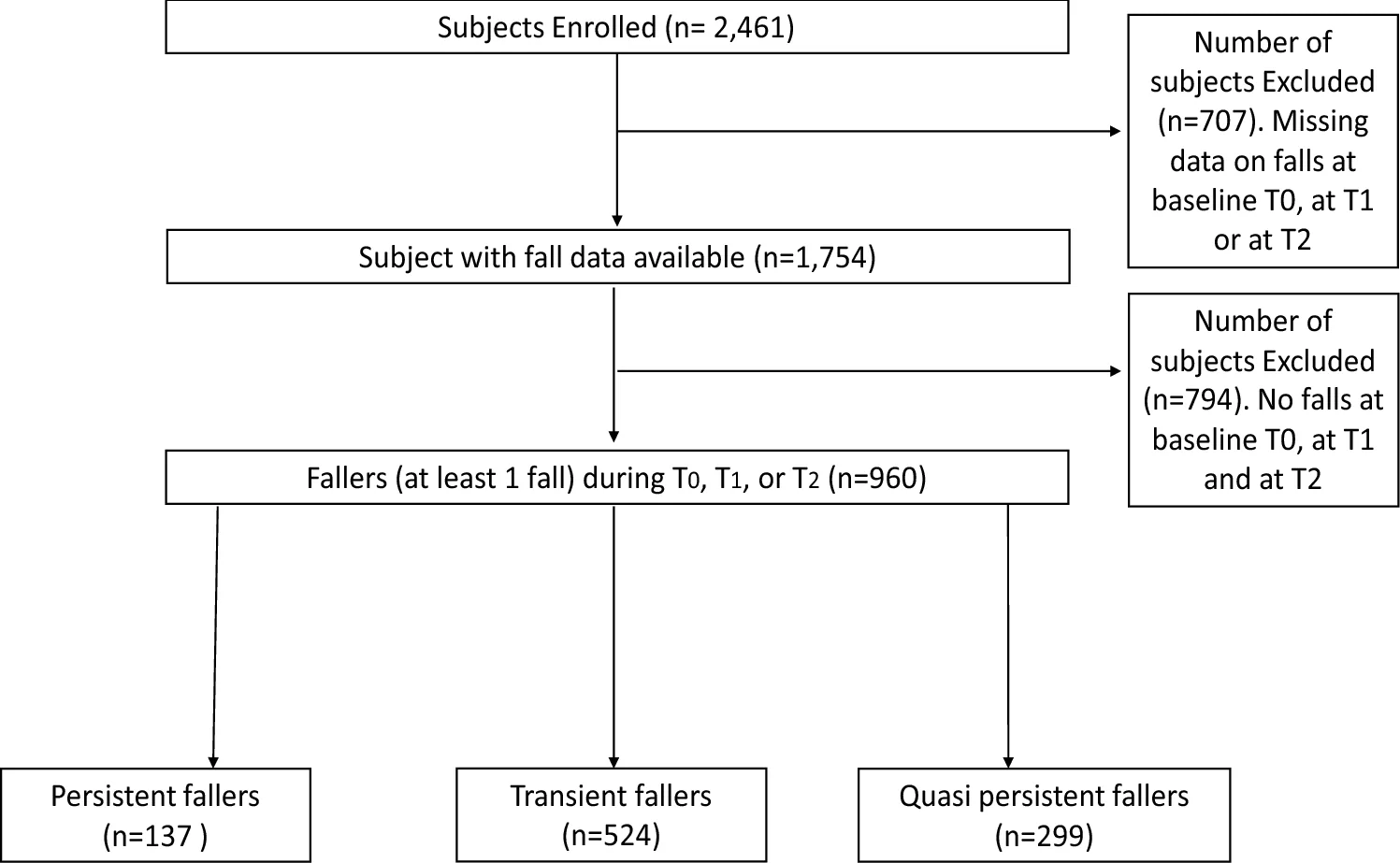
Study flowchart

Of the 2461 older adults initially enrolled in the study, 707 were excluded due to missing data on falls at T0, T1 and T2. In addition, 794 older adults were excluded, because they reported no falls at any of the three assessments (flowchart, Fig. 1). Consequently, 960 older adults who reported at least one fall during T0, T1 and T2 were grouped into P-Fallers (*n* = 137, 14.3%), Q-Fallers (*n* = 299, 31.1%) and T-Fallers (*n* = 524, 54.6%). In total, 2,508 falls (P-Fallers 894, Q-Fallers 919, T-Fallers 919) were analyzed in this study.

### Statistical analysis

Demographics, medical condition, and functional status of the overall study population were reported among P-Fallers, Q-Fallers and T-Fallers at baseline. Continuous variables were all expressed as median and interquartile range (IQR) according to their not normal distribution assessed through visual inspection and Kolmogorov–Smirnov test. Categorical variables were presented as absolute and relative (%) frequencies. Non-parametric Kruskal–Wallis tests with Bonferroni adjustment for post-hoc comparisons and chi-squared tests were used to assess the differences in fall characteristics between the three groups at baseline. Fall circumstances, locations, causes, and consequences (i.e., severity of injurious falls) are presented as percentages of the total number of falls in each group across the entire study period (i.e., T0, T1 and T2). This approach ensures that the analysis accurately reflects the specific circumstances and consequences of each individual fall throughout the study period.

Finally, a multinominal logistic regression model was performed to study the probability of being a P-Faller vs. T-Faller as well as P-Faller vs. Q-Faller using baseline data. Possible correlates like SPPB, CIRS, number of medications, eGFR (BIS equation), LUTS, Hemoglobin (g/dl), Euro-Qol 5D, age and sex, were first examined in crude logistic regression models and after in a fully adjusted model. Both models were adjusted for the country, where the study center was located. Statistical significance was set a priori at *p* < 0.05. All analyses were performed with SPSS v.24 (SPSS Inc., Chicago, IL, USA).

## Results

### Medical and functional characteristics

Descriptive statistics characterizing the three groups at baseline (T0), P-Fallers, T-Fallers, and Q-Fallers, are presented in Table 1. Groups were similar in age, sex, marital status and BMI; however, P-Fallers exhibited a consistently worse overall health status. P-Fallers had a significantly higher CIRS score (10.0 vs. 8.0 and 8.0, respectively), were more likely to be taking five or more medications (74.5% vs. 64.7% and 70.9%, respectively), and more often reported moderate to severe LUTS (49.6% vs. 28.6% and 33.4%, respectively). P-Fallers had significantly lower hemoglobin levels (13.3 g/dl vs. 13.5 g/dl and 13.6 g/dl, respectively), lower SPPB scores (8.0 vs. 9.0 and 10.0, respectively) than Q-Fallers and T-Fallers. P-Fallers also had a significantly lower EQ-5D Visual Analogue Scale (VAS) scores as well as worse EuroQol 5D index scores compared to T-Fallers (70.0 vs. 75.0, and 9.0 vs. 7.0).

**Table 1:** Baseline characteristics of persistent fallers, transient fallers and quasi persistent fallers and number of falls during the whole study period

Variable	Group A Persistent fallers (*n* = 137)	Group B Quasi persistent (*n* = 299)	Group C Transient fallers (*n* = 524)	*p* value	Post-hoc
Falls (total number), *n*	894	919	695		
Falls per participant, median (IQR)	6.0 (4.5)	2.0 (2.0)	1.0 (0.0)	< 0.001	a vs. b; a vs. c;
Participants experiencing fall with fracture, *n* (%)	29 (21.2)	50 (16.7)	71 (13.5)	0.075	
More than 1 fall within 1 year, *n* (%)	137 (100.0)	299 (100.0)	111 (21.2)	< 0.001	a vs. c; b vs. c;
Age (years), median (IQR)	79.0 (5.0)	79.0 (7.0)	79.0 (6.0)	0.385	
Sex: female, *n* (%)	86 (62.8)	190 (63.5)	311 (59.4)	0.452	
BMI (kg/m2), median (IQR)	27.7 (5.8)	27.3 (5.9)	27.3 (5.5)	0.579	
Marital status, *n* (%)					
Single	10 (7.3)	24 (8.0)	35 (6.7)	0.152	
Married/living with a partner	71 (51.8)	138 (46.2)	278 (53.1)		
Separated/divorced	13 (9.5)	14 (4.7)	33 (6.3)		
Widowed	43 (31.4)	123 (41.1)	178 (34.0)		
Education (years), median (IQR)	12.0 (6.0)	12.0 (7.0)	12.0 (7.0)	0.560	
Diabetes, *n* (%)	41 (29.9)	68 (22.7)	123 (23.5)	0.229	
Hypertension, *n* (%)	110 (80.3)	229 (76.6)	389 (74.2)	0.315	
Stroke, *n* (%)	10 (7.3)	24 (8.0)	29 (5.5)	0.355	
Hip Fractures, *n* (%)	10 (7.3)	19 (6.4)	29 (5.5)	0.715	
COPD, *n* (%)	23 (16.8)	34 (11.4)	57 (10.9)	0.155	
Osteoporosis, *n* (%)	40 (29.2)	110 (36.8)	169 (32.3)	0.230	
Parkinson’s disease, *n* (%)	4 (2.9)	8 (2.7)	7 (1.3)	0.288	
Anemia, *n* (%)	34 (25.6)	50 (18.1)	88 (17.6)	0.103	
CKD (BIS < 60), *n* (%)	88 (64.2)	188 (63.1)	305 (58.3)	0.263	
eGFR (BIS equation) (ml/min/1,73 m2), median (IQR)	54.7 (19.8)	54.9 (22.9)	57.2 (16.7)	0.075	
CIRS (total score), median (IQR)	10.0 (7.0)	8.0 (7.0)	8.0 (6.0)	< 0.001	a vs. b; a vs. c
Take ≥ 5 current medications, *n* (%)	102 (74.5)	212 (70.9)	339 (64.7)	0.041	a vs. c; b vs. c
Moderate/severe LUTS, *n* (%)	68 (49.6)	94 (31.4)	150 (28.6)	< 0.001	a vs. b; a vs. c
Hemoglobin (g/dl), median (IQR)	13.3 (2.1)	13.5 (2.0)	13.6 (1.8)	0.021	a vs. b; a vs. c
Hand grip strength (kg), median (IQR)	23.0 (11.0)	22.0 (12.0)	24.0 (13.0)	0.061	
GDS > 5, *n* (%)	26 (19.0)	45 (15.1)	69 (13.2)	0.221	
MMSE < 24, *n* (%)	12 (8.8)	20 (6.7)	44 (8.4)	0.632	
SPPB (total score), median (IQR)	8.0 (4.0)	9.0 (5.0)	10.0 (4.0)	< 0.001	a vs. c; b vs. c
SPPB (balance score), median (IQR)	3.0 (2.0)	3.0 (2.0)	4.0 (1.0)	< 0.001	a vs. c; b vs. c
SPPB (gait score), median (IQR)	3.0 (2.0)	3.0 (1.0)	4.0 (1.0)	0.001	a vs. c; b vs. c
Fear of falling not at all concerned (at least 1), *n* (%)	80 (58.4)	172 (57.5)	275 (52.5)	0.253	
EQ-visual analogue scale (EQ-5D VAS), median (IQR)	70.0 (30.0)	70.0 (30.0)	75.0 (25.0)	0.001	a vs. c; b vs. c

Values are *n* (%) for categorical variables, and median (interquartile range) for continuous

*BMI* Body mass index, *CIRS* Cumulative illness rating score, *CKD* Chronic kidney disease, *COPD* Chronic obstructive pulmonary disease, *eGFR* Estimated glomerular filtration rate, *GDS* Geriatric depression scale, *LUTS* Lower urinary tract symptoms, *MMSE* Mini-mental state examination, *SPPB* Short physical performance battery, *QoL* Quality of life, *VAS* Visual analogue scale

Although differences were not statistically significant, P-Fallers consistently had higher prevalence of diabetes, hypertension, stroke, hip fractures, COPD, Parkinson’s disease, anemia, have reduced kidney function and lower handgrip strength compared to T-Fallers (Table 1), i.e., a worse overall health status.

### Circumstances of falls

**Fig. 2:**
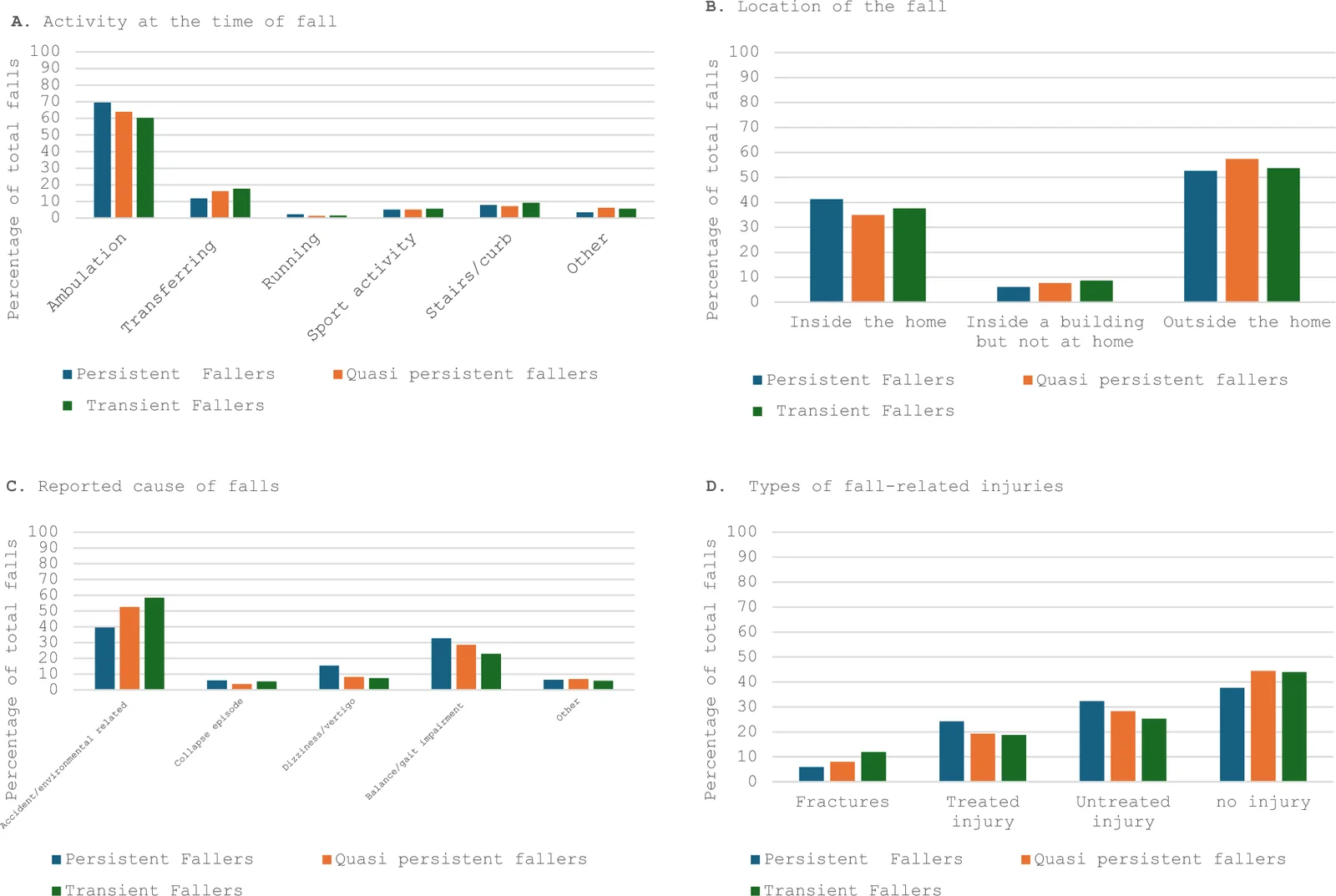
Circumstances of falls according to persistent fallers, transient fallers and quasi persistent fallers are presented as the percentage of self-reported cases for: **A** activity at the time of fall; **B** location of the fall; **C** reported causes of falls; **D** type of fall injuries

Findings regarding the circumstances of falls are presented in Fig. 2A–D. Due to missing data, different case numbers are available for location of falls (*n* = 2067), activity during falls (*n* = 2065), causes of falls (*n* = 2064), and consequences of falls (*n* = 2058). Figure 2A shows that among P-Fallers ambulation was ranked as the top activity that accounted for falls, higher than T-Fallers and Q-Fallers (69.5% vs. 60.3% and 63.9%, respectively). Transferring was the second most common activity that accounted for falls; however, it was lower among P-Fallers compared to T-Fallers and Q-Fallers (11.8% vs. 17.6% and 16.2%, respectively). Figure 2B shows that falls in general were more likely to occur outdoors rather than at home (52.6–57.4% vs. 34.9–41.3%, respectively). P-Fallers experienced more falls at home compared to T-Fallers and Q-Fallers (41.3% vs. 37.6% and 34.9%, respectively) while falling less frequently outdoors (52.6% vs. 53.7% and 57.4%, respectively; Fig. 2B). The most frequently cited causes of falls are summarized in Fig. 2C. Among P-Fallers, 39.5% of falls were due to external or environmental factors that caused trips or slips or collisions with objects, compared to 58.4% among T-Fallers. Balance and gait impairments were attributed to 32.7% of falls among P-Fallers and 15.4% to dizziness, compared to 22.9% and 7.4%, receptively among T-Fallers. As illustrated in Fig. 2D, 5.9% of falls among P-Fallers resulted in bone fractures compared to 11.9% among T-Fallers, while treated and untreated injuries were higher in P-Fallers than in T-Fallers (24.2 + 32.3% vs. 18.8 + 25.3%, respectively).

We examined the probability of being a P-Faller, using crude multinominal logistic regression models first, and a fully adjusted model after (Table 2). No significant associations were found for age, sex, and eGFR. The SPPB total score was significant at crude level, suggesting lower physical performance among P-Fallers, but lost significance after adjustment. In addition, number of medications and hemoglobin levels, were significant at univariate level, but interestingly both were not significant after adjustment. The fully adjusted model show that higher comorbidity burden (i.e., CIRS total score) and LUTS symptoms were significantly associated with persistent falling (OR = 1.11; 95% CI 1.06–1.17; and OR = 1.92; 95% CI 1.26–2.93, respectively), indicating that those with worse medical conditions were more likely to experience repeated falls. In addition, poorer self-rated health at baseline (EuroQol 5D index scores) was significantly associated with the probability of being a P-Faller (OR = 1.09; 95% CI 1.02–1.17). Multinominal logistic regression of the probability of being a P-Faller compared to a Q-Faller revealed similar results, with only LUTS (OR = 1.82; 95% CI 1.17–2.82) remaining significant at the fully adjusted level (Table 2).

**Table 2:** Multinomial logistic models (reference category: persistent fallers; all models adjusted for country)

	Transient fallers vs. persistent fallers		Quasi persistent fallers vs. persistent fallers	
	Crude model	Fully adjusted model	Crude model	Fully adjusted model
	OR (95% CI)		OR (95% CI)	
Age (years)	0.99 (0.95–1.04)	–	1.02 (0.97–1.07)	–
Sex (female)	0.87 (0.59–1.28)	–	1.03 (0.68–1.57)	–
SPPB total score*	1.15 (1.08–1.23)	1.09 (1.01–1.19)	1.05 (0.98–1.12)	0.99 (0.92–1.09)
SPPB balance score	1.31 (1.12–1.53)	–	1.05 (0.89–1.23)	–
SPPB gait score	1.36 (1.14–1.62)	–	1.15 (0.96–1.38)	–
CIRS total score*	0.88 (0.84–0.92)	0.94 (0.89–0.99)	0.92 (0.88–0.96)	0.95 (0.90–1.01)
Take ≥ 5 current medications*	0.63 (0.41–0.96)	1.30 (0.78–2.20)	0.84 (0.53–1.32)	1.39 (0.80–2.41)
Moderate/severe LUTS*	0.41 (0.28–0.60)	0.54 (0.35–0.83)	0.46 (0.31–0.70)	0.57 (0.36–0.90)
eGFR BIS equation (ml/min/1,73 m^2^)	1.01 (1.00–1.03)	–	1.01 (0.99–1.02)	–
Hemoglobin (g/dl)*	1.18 (1.03–1.35)	1.11 (0.96–1.29)	1.16 (1.01–1.35)	1.15 (0.98–1.34)
Euro-Qol 5D questionnaire*	0.85 (0.80–0.90)	0.90 (0.84–0.97)	0.91 (0.86–0.96)	0.94 (0.87–1.01)
EQ-5D VAS	1.02 (1.01–1.03)	–	1.01 (0.99–1.02)	–

*CI* Confidence interval, *OR* Odds ratio, *CIRS* Cumulative illness rating score, *eGFR BIS* Estimated glomerular filtration rate Berlin initiative study, *LUTS* Lower urinary tract symptoms, *SPPB* Short physical performance battery, *QoL* Quality of life, *VAS* Visual analogue scale

^*^variables used in fully adjusted model

## Discussion

Our findings show that the majority of falls among independently living, older adults occurred during ambulation which is consistent with previous reports [10–22, 25–28]. The highest prevalence of falls during ambulation was observed among P-Fallers who exhibited also a lower prevalence of falls during transfers than the other groups which may limit the activities of daily living. Interestingly, P-Fallers also reported a higher prevalence of balance and gait impairments and dizziness as the cause of their falls, compared to T-Fallers and Q-Fallers. In line with the self-reports, P-Fallers also demonstrated significantly lower SPPB scores, indicating objective reduction in balance and gait performance, and supporting the highest fall risk. Consequently, they may limit the time spent outside the home, which could explain their lower prevalence of outdoor falls compared to T-Fallers and Q-Fallers. However, these mobility behaviours were not evaluated in this study and have, therefore, to be verified in future trials. At home P-Fallers experienced more falls compared to the other two groups. Since falls at home tend to be less vigorous than those occurring outdoor, this may also explains why P-Fallers had a lower likelihood of sustaining a fracture after a fall, compared to T-Fallers and Q-Fallers.

Interestingly, falls caused by environmental factors such as trips and slips are more frequent among T-Fallers and Q-Fallers compared to P-Fallers. We speculate that although age-related deterioration in balance function increases fall risk in all older adults, T-Fallers may overestimate their abilities. This might lead to riskier behavior and a higher incidence of outdoor falls and falls due to external or environmental factors [29, 30]. Higher outdoor activity levels and greater exposure to fall opportunities may then contribute to the elevated chance-related risk of falls [31]. Previous research has also shown that older adults tend to fall in locations, where they spend the most time [11], thus indoor falls are more common among individuals with worse overall health status and less active lifestyles [12–14, 22, 32–35]. A greater proportion of robust older adults tend to experience higher outdoor falls caused by environmental factors. Outdoor falls are often more forceful, which likely explains why a higher proportion of falls led to fractures in this group [12, 13, 22, 35]. Consistent with that we found that the rate of fall-related bone fractures was twice as high among T-Fallers as among P-Fallers, despite the former generally exhibiting better health and balance status.

Previous reports revealed that those who had fallen outdoors had better health characteristics, whereas those who had fallen indoors were generally in worse overall health status [15, 22]. This is consistent with our findings, which show that compared to T-Fallers, P-Fallers are generally worse overall health status, reflected by an higher prevalence of LUTS and higher CIRS (see Tables 1 and 2). However, physical function measured by SPPB was not different between T- and P-Fallers in the fully adjusted model. Therefore, the increased risk of falls seems to be primarily driven by a declining general health status that secondarily leads to a worsening physical functionality. These findings highlight the importance of targeting comorbidity management, reinforcing the relevance of health status in fall risk.

The present findings suggest that fall characteristics and circumstances differ between the three subgroups of community-dwelling older adult fallers: P-Fallers, Q-Fallers, and T-Fallers. Data suggest that while improving balance and gait function is important specifically for P-fallers, all groups can benefit from such interventions [36]; however, T-Fallers in particular may benefit from increased awareness of their balance reactive abilities and attention to environmental hazards. While such interventions may not improve balance, accurate self-assessment of motor skills is a distinct and crucial capability for effective fall prevention as it may prevent an overestimation of balance function, thereby decreasing the likelihood of injurious falls. Although clinical tests of balance show higher balance performance among T-Fallers, these tests may not able to capture impaired balance reactive abilities. Previous research [37–42] has shown that age-related decline in balance reactive responses affects all older adults, including those who have not fallen yet. These reactive balance responses can be improved through a specific reactive balance training that specifically trains the ability to respond effectively to unexpected balance loss, such as trips or slips [43–47], and has been shown to reduce fall incidence among older adults [48, 49]. Therefore, fall prevention programs should not only target high-risk populations such as P-Fallers but also include T-Fallers and Q-Fallers, incorporating personalized interventions tailored to each individual's actual abilities. This is further supported by recent findings demonstrating that fall prevention strategies should include the promotion of physical activity [50]. Moreover, their evidence indicated that injurious falls are associated with subsequent reductions in physical activity [50]. This underscores the importance of targeted interventions aimed at maintaining or restoring activity levels following a fall, particularly when injuries are involved.

An interesting finding emerged regarding Q-Fallers, who appeared to share characteristics with both P-Fallers and T-Fallers, as if they had not fully aligned with either group. In several aspects of their fall profiles such as activities during the fall, causes, and resulting injuries, as well as their SPPB scores they consistently fell between P-Fallers and T-Fallers. This is in line with the WFP algorithm [7] which extended the former algorithm exactly with moderate fall risk group.

This study has several limitations. First, fall data were primarily based on retrospective 12-month recall at baseline, 12, and 24 months or obtained from medical sources, which may have led to under-reporting, especially of minor or non-injurious falls, due to recall bias. Second, the sample consisted of relatively healthy, community-dwelling older adults, limiting generalizability to frailer or institutionalized populations. Third, we obtained CaF with a one item single question and not with the more comprehensive and validated Falls Efficacy Scale International (FES-I) questionaire. However, a recent systematic review has shown the reliability of such single item question as being predictive of future falls [51]. Fifth, daily step counts and mobility behavior were not measured, preventing calculation of fall rates relative to activity levels and causal analysis of the found fall characteristics. Sixth, selection bias may also exist, as participation was voluntary. Nonetheless, the study’s large, multinational sample and engaged participants and using novel analyses between P-Fallers, T-Fallers and Q-Fallers, enhance its value and likely improved data accuracy.

In conclusion, this study defines distinct faller profiles: Persistent-Fallers often have clinically proven balance and gait impairments, fall indoors, but sustain fewer fractures per fall and are more strongly linked to overall medical burden; Transient-Fallers are generally healthier, fall outdoors more often, and have higher fracture rates per fall; and Quasi-Persistent Fallers exhibit intermediate characteristics, showing early decline, medically between P- and T-Fallers. P-Fallers likely represent a consistently high-risk group with ongoing vulnerability, while T-fallers may reflect temporary health impairments or situational risk. Quasi-persistent fallers appear to have fluctuating risk influenced by short-term medical conditions or modifiable environmental factors. According to the WFP algorithm [7], P-Fallers correspond to the high-risk group warranting comprehensive geriatric assessment and accordingly treatment, including balance training. Q-Fallers align with the moderate-risk group and may benefit from targeted exercise interventions. T-Fallers fit the lower-risk group and may benefit from education or physical activity advice. These findings highlight the need for future studies to determine whether tailored fall-prevention strategies designed for the distinct faller profiles, such as improving caution and balance in T-Fallers, enhancing function and medical management in P-Fallers, and addressing fall risk in Q-Fallers to prevent progression, can effectively reduce fall rates.

## Data Availability

Data will be available for SCOPE researchers through the project website (www.scopeproject.eu).

## Abbreviations

CaF: Concerns about falling
EQ-VAS: EQ-visual analogue scale
eGFR: Glomerular filtration rate
BIS: Berlin initiative study equation
GDS: Geriatric depression scale
CIRS: Cumulative illness rating scale
OR: Odds ratio
